# Psychopathic traits are associated with reduced attention to the eyes of emotional faces among adult male non-offenders

**DOI:** 10.3389/fnhum.2015.00552

**Published:** 2015-10-07

**Authors:** Steven M. Gillespie, Pia Rotshtein, Laura J. Wells, Anthony R. Beech, Ian J. Mitchell

**Affiliations:** School of Psychology, University of BirminghamBirmingham, UK

**Keywords:** psychopathy, eye gaze, attention, emotion, facial expression recognition

## Abstract

Psychopathic traits are linked with impairments in emotional facial expression recognition. These impairments may, in part, reflect reduced attention to the eyes of emotional faces. Although reduced attention to the eyes has been noted among children with conduct problems and callous-unemotional traits, similar findings are yet to be found in relation to psychopathic traits among adult male participants. Here we investigated the relationship of primary (selfish, uncaring) and secondary (impulsive, antisocial) psychopathic traits with attention to the eyes among adult male non-offenders during an emotion recognition task. We measured the number of fixations, and overall dwell time, on the eyes, and the mouth of male and female faces showing the six basic emotions at varying levels of intensity. We found no relationship of primary or secondary psychopathic traits with recognition accuracy. However, primary psychopathic traits were associated with a reduced number of fixations, and lower overall dwell time, on the eyes relative to the mouth across expressions, intensity, and sex. Furthermore, the relationship of primary psychopathic traits with attention to the eyes of angry and fearful faces was influenced by the sex and intensity of the expression. We also showed that a greater number of fixations on the eyes, relative to the mouth, were associated with increased accuracy for angry and fearful expression recognition. These results are the first to show effects of psychopathic traits on attention to the eyes of emotional faces in an adult male sample, and may support amygdala based accounts of psychopathy. These findings may also have methodological implications for clinical studies of emotion recognition.

## Introduction

In his seminal description of the psychopathy syndrome, Cleckley (1976) refers to a subgroup of hospitalized patients who, despite appearing as otherwise overtly “normal,” and free from insanity or delusion, were characterized by severe emotional detachment, callousness, a lack of remorse, or guilt, and high levels of superficial charm. This description is still widely relevant to modern day descriptions of the psychopathic personality, with many of these criteria being adapted for use in the Psychopathy Checklist—Revised [PCL-R] (Hare, 1991, 2003). According to the PCL-R, the psychopathy construct is underpinned by two correlated factors, Factor 1 and Factor 2. Factor 1 taps the interpersonal/affective features of the disorder, including a callous lack of empathy, a cunning, and manipulative interpersonal style, and a lack of remorse, or guilt, while Factor 2 measures the behavioral/lifestyle features including anti-social behavior, irresponsibility, and poor behavioral control.

Although antisocial behavior represents a defining feature of the psychopathy construct (Hare and Neumann, 2010), the presence of the core affective features of the disorder differentiates it from other syndromes characterized by marked levels of antisociality, criminality, and aggression. For example, antisocial behavior with psychopathic tendencies has been successfully distinguished from more generally antisocial behavior in terms of emotion processing (Kosson et al., 2006; Verona et al., 2012), brain structure (Gregory et al., 2012), and heritability among children at age seven (Viding et al., 2005) and nine (Viding et al., 2008). Psychopathy therefore refers to a distinct subgroup of antisocial individuals characterized by heritable deficits in the processing of emotional stimuli.

Offenders with psychopathy are also distinguishable from non-psychopathic offenders on the basis of emotional expression recognition, with psychopaths showing marked impairments in the recognition of fearful face affect (Blair et al., 2004). Although these findings have been confirmed in the meta-analysis of Marsh and Blair (2008), others suggest more pervasive impairments including deficits for expressions of sadness and disgust (Kosson et al., 2002; Dolan and Fullam, 2006; also see Dawel et al., 2012). Similar to adult studies, callous-unemotional (CU) traits in developmental samples have also been linked with impaired recognition of sad and afraid facial expressions (Blair et al., 2001; Muñoz, 2009), and afraid bodily postures (Muñoz, 2009). However, impairments in judging fear face affect may be gender specific. For example, female psychopathic offenders, relative to non-psychopathic inpatient controls, showed particular deficits for sad, neutral, and surprise, but not fearful expressions (Eisenbarth et al., 2008). Blair (1995, 2001) argues that psychopathy related deficits in emotional expression recognition, and difficulties in experiencing others submissive displays as aversive, may result in an increased risk of instrumental or proactive aggression.

As well as research with clinical and forensic samples, research examining the cognitive, affective, and functional correlates of psychopathic traits in the general population has received growing attention. Research with both non-clinical adult male and adult female samples has revealed expected correlations of self-reported psychopathic traits with both empathy and morality (Seara-Cardoso et al., 2012, 2013), while psychopathic traits in a healthy college student sample were related to a particular deficit in the recognition of fearful expressions only in the frontal view (Montagne et al., 2005). These findings are consistent with results showing that psychopathic traits are normally distributed in the general population (Hare and Neumann, 2008), and that psychopathy is better understood as a dimensional construct rather than as a taxonomy (Edens et al., 2006; Guay et al., 2007). Seara-Cardoso and Viding (2014) also note that functional neuroscience findings from the general population appear to closely mirror those from clinical samples, supporting the conclusion that individual differences in self-reported psychopathic traits relate to individual differences in brain function (Seara-Cardoso and Viding, 2014). Thus, although clinically elevated levels of psychopathic personality may be rare in the general population, continuities in the mechanisms underlying psychopathic personality nonetheless exist.

Particular support for continuum based models of psychopathy also exists with respect to emotional expression recognition. For example, Blair and Coles (2000) showed that accuracy for sad and fearful faces is inversely associated with both the CU and impulsivity/conduct features of psychopathy. Similarly, Prado et al. (2015) found significant inverse correlations of primary psychopathic traits, characterized by a lack of empathy and remorseless use of others, with accuracy for anger, fear, sad, shame, and disgust expressions. However, these authors found no relationship of emotion recognition with secondary psychopathic traits, characterized by impulsivity and recklessness (Prado et al., 2015).

The precise mechanisms underlying psychopathy related impairments in emotion recognition remain unclear. However, functional imaging experiments with children with CU traits have revealed hypoactivity of the amygdala, a crucial structure for the processing of fear related information, in response to images of fearful faces (Marsh et al., 2008; Jones et al., 2009), and in response to masked images of fearful eyes (Viding et al., 2012). Furthermore, in a large adult sample of male criminals assessed on the PCL-R, Decety et al. (2014) found reduced amygdala response to fearful and sad faces among psychopaths relative to non-psychopaths. These results suggest that impairments in fear face affect recognition in psychopathy may reflect dysfunction of the amygdala. This implication is supported by studies showing that patients with bilateral amygdala dysfunction also show difficulties in recognizing fearful facial expressions of emotion (Adolphs et al., 1994; Calder, 1996; Broks et al., 1998).

One mechanism for impaired fearful face recognition may involve reduced attention to the eye region of emotional faces. For example, Adolphs and colleagues showed that SM, a single patient with amygdala dysfunction, made less use of information from the eye region relative to controls, and failed to fixate the eye region of fearful, as well as other emotional expressions (Adolphs et al., 2005). When instructed to fixate the eye region, SM acquired normal levels of performance (Adolphs et al., 2005). The importance of attention to the eyes for fear recognition is emphasized by results showing that the amygdala is highly responsive to information from the eye region (Whalen et al., 2004), that reflexive shifts of attention toward the eye region are correlated with amygdala activity (Gamer and Büchel, 2009), and that information from the eyes is particularly important for recognizing fearful expressions (Smith et al., 2005).

On the basis of these findings, it has been hypothesized that impaired fear recognition in psychopathy may stem from a breakdown in the directing of attention toward the eye region. Consistent with this hypothesis, temporary correction of emotion recognition impairments has been observed among children with antisocial behavior and CU traits following instructions to fixate the eyes of emotional faces (Dadds et al., 2006). Moreover, CU traits among 100 boys ranging in age from 8 to 15 years (mean age = 12.4 years) are associated with fewer and shorter fixations of the eye region during an expression recognition task, and accuracy of fearful face recognition is positively correlated with both the number, and length, of fixations on the eye region (Dadds et al., 2008).

However, work on the relationship of psychopathic tendencies with attention to the eyes has specifically focused on the association with CU traits in a developmental sample, and similar relationships are yet to be demonstrated in adult samples of offenders or non-offenders. Furthermore, impaired emotion recognition has been noted in relation to generally antisocial and violent offending (Hoaken et al., 2007; Robinson et al., 2012; Bagcioglu et al., 2014; Gillespie et al., 2015), and in relation to the lifestyle/antisocial features of psychopathy (Blair and Coles, 2000). As such, a failure to fixate the eye region may not be specific to the core affective features of psychopathy.

The importance of considering the separable dimensions of the psychopathy construct have been highlighted by Hicks and Patrick (2006), who observed the presence of suppressor effects in the ways in which PCL-R Factors 1 and 2 relate to negative emotionality in offending samples. As summarized by Hicks and Patrick (2006), suppressor effects occur where two correlated predictors show opposing relations with a criterion variable. When accounting for potential suppressor effects, Hicks and Patrick demonstrated convincing evidence for a negative association of Factor 1 with negative emotionality, including distress, fear, and anger, while positive associations were found in relation to Factor 2. Other examples of suppressor effects in relation to discrete psychopathic traits have also been highlighted by Blonigen et al. (2010), Vanman et al. (2003), and Lockwood et al. (2013).

In the current experiment, we aimed to investigate the influence of primary and secondary psychopathic traits on accuracy of expression recognition, as well as on eye scan paths for emotionally expressive faces in an adult male, non-offending sample. Although psychopathy is typically assessed in adult male offenders using the PCL-R, psychopathic tendencies may also be assessed among offenders, and non-offenders, using self-report personality scales. These measures typically assess psychopathy along similar dimensions to the PCL-R, and include reference to the interpersonal/affective, and lifestyle/antisocial features of the disorder. We measured attention toward two main regions of interest (ROIs), the eyes and the mouth, on the basis that these represent the two most emotionally salient aspects of the face (Eisenbarth and Alpers, 2011). We used eye tracking techniques to measure both total dwell time, and the total number of fixations, on each region.

Eye scan paths for emotional faces vary as a function of the emotional content of the expression (Eisenbarth and Alpers, 2011), and so in this study we used images of the six “basic” expressions: anger, disgust, fear, happy, sad, and surprise. Further, we manipulated the intensity of these expressions for two reasons. Firstly, ambivalent expressions are more representative of facial expressions outside of the laboratory and hence have higher ecological validity; and secondly, this has been shown to make the task more sensitive to subtle differences in the processing of facial expressions (Calder et al., 1996; Adolphs and Tranel, 2004). We also considered the sex of the face displaying the expression, using both male and female models, with emotion recognition found to vary as a function of the sex of the model conveying the expression (Tucker and Riggio, 1988; Hess et al., 1997). Furthermore, psychopathic traits have been found to manifest differently for males and females (Coid et al., 2009), and some evidence suggests that male and female participants show differences in eye scan paths for emotional expressions (Hall et al., 2010). As such, recruitment was limited to male participants only.

We predicted that primary, but not secondary, psychopathic traits would be associated with impaired fear expression recognition, and a reduced tendency to direct attention toward the eye region of emotional faces. These predictions are consistent with the finding that primary, but not secondary, psychopathic traits are related to impairments in fearful face affect recognition in a sample of non-offenders (Prado et al., 2015). We made no specific predictions about the relationship between psychopathic traits and the intensity of the expression shown, or the sex of the model displaying the expression.

## Methods

### Participants

A total of 38 participants were recruited from the staff and student population of the University of Birmingham. All participants were male and ranged in age from 19 to 39 years (*M* = 23.2, *SD* = 4.9). The majority of participants were white Caucasian (*n* = 32). All participants had normal or corrected to normal vision. Ethical approval for the study was granted by the University of Birmingham, UK, Committee for Ethical Review for Science, Technology, Engineering, and Mathematics (STEM). All participants signed their fully informed consent.

### Materials

#### Facial expression stimuli

**Figure 1:**
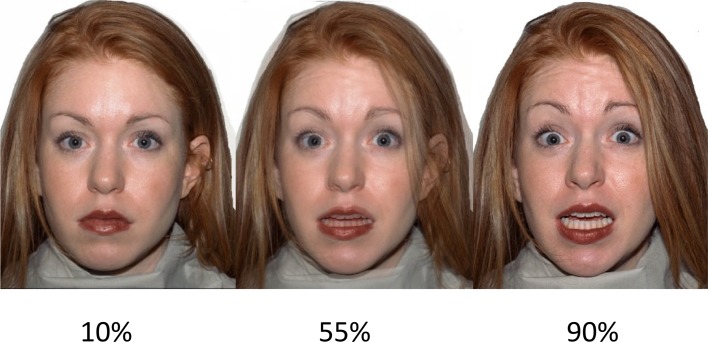
**Example stimuli**. A female fearful expression expressed at (left–right): low intensity (10%), moderate intensity (55%), and high intensity (90%). Reproduced from Gillespie et al. (2015).

Ten different Caucasian models (five females) were selected from the NimStim Face Stimulus Set (Tottenham et al., 2009; http://www.macbrain.org/resources.htm). Each model conveyed a neutral expression, and each of the six basic emotions: anger, disgust, fear, happy, sad, and surprise. The models were selected on the basis of validity data that indicate a high mean proportion correct for the chosen expressions: neutral (*M* = 0.84, *SD* = 0.13), angry (*M* = 0.85, *SD* = 0.83), disgust (*M* = 0.85, *SD* = 0.13), fear (*M* = 0.84, *SD* = 0.13), happy (*M* = 0.85, *SD* = 0.13), sad (*M* = 0.85, *SD* = 0.13), surprised (*M* = 0.85, *SD* = 0.13). In order to manipulate the intensity of the emotional expressions, each expression was morphed from neutral to 100% expressive in 10 successive frames using the STOIK Morph Man software (http://www.stoik.com/products/video/STOIK-MorphMan/). This resulted in 10 morphed continua for each of the six expressions for the 10 selected models. For task purposes, three frames of varying intensity were selected for each expression; mild intensity (10% expressive); moderate intensity (55% expressive); high intensity (90% expressive). See Figure 1 for example stimuli. Thus, we had 18 faces across all expressions for each model, 180 faces in total.

#### Levenson self-report psychopathy scales [LSRP]

The LSRP (Levenson et al., 1995) was developed for the assessment of psychopathic traits in non-institutionalized populations. The LSRP contains 26 items measured on a four-point Likert scale. While 16 items measure the primary characteristics associated with psychopathic personality, including selfishness and a lack of care for others, the remaining items tap secondary traits, and includes proneness to boredom and impulsivity. Although the labels “primary” and “secondary” are more commonly associated with variants of psychopathy that differ in the experience of anxiety, the two-factor structure of the LSRP is supported by evidence of medium sized correlations of the primary and secondary subscales with Factors 1 and 2 of the PCL-R, respectively (Brinkley et al., 2001). Levenson et al. have demonstrated adequate internal validity of the LSRP in a student sample, with a Cronbach's alpha of 0.82 for the primary subscale, and 0.63 for the secondary subscale. Levenson et al. (1995) considered this to be a reliable estimate for a 10 item scale. One participant failed to complete the LSRP.

#### Eye tracking

We used an EyeLink 1000 eye tracking system (SR Research Ltd.) to record eye gaze and dwell time. Although viewing was binocular, only movements of the right eye were recorded. Gaze location was sampled once every millisecond. Participants were tested using either a head mounted eye tracking system, or a towered eye tracker. In the analysis of data we test for potential differences in the eye tracking data recorded using these different techniques.

#### Procedure

Participants were seated at a desk with a computer monitor and keyboard. Participants whose eye movements were tracked using the eye tracking tower were supported by a chin-rest and a head-rest in order to minimize head movements. Where participants were tested using a head mounted eye tracking system, the head mount was placed on the participants head and adjusted to fit. We first calibrated the eyes using a standard calibration procedure with nine fixation points. A validation procedure was also completed to ensure accuracy of the recording equipment. Facial expression stimuli were presented on a computer monitor in a randomized order. At the start of each trial, the experimenter confirmed that the participants' eye gaze fell on the fixation point. For each trial, participants were asked to categorize the expression as either neutral or one of the six basic emotions: anger, disgust, fear, happy, sad, or surprise, using the keys 0–6, respectively, on a computer keyboard. The expression labels and relevant number keys were listed on the left hand side of the screen. Stimuli remained on-screen until a response was made. There were 180 trials in total, each presenting a different stimulus that varied in model, expression, and intensity. The experimental procedure was developed using EyeLink experiment builder (SR Research, Ltd.).

### Data analysis

#### Behavioral data

Accuracy data were available for 28 participants; accuracy data for the remaining participants was not recorded due to a technical error. The available accuracy data were analyzed using a six (emotion) × three (intensity) × two (sex of face) mixed ANCOVA. We used primary and secondary psychopathy scores as between subject covariates. Note that by including both primary and secondary traits in the same ANCOVA model, we ensure that any effects observed for one sub-scale are controlled for and hence beyond the effect of the other sub-scale. We therefore account for potential suppressor effects, as identified by Hicks and Patrick (2006), between the primary and secondary features of the psychopathy construct. Based on a-priori hypotheses, we also computed zero-order and partial correlations for recognition accuracy of the moderate and high intensity negative expressions, with scores on the primary and secondary psychopathy scales.

#### Eye tracking data

The analysis of eye scan paths focused on pre-determined regions of interest (ROI), specifically the eye and mouth regions. The eye region was defined using a rectangle of 289 × 100 pixels including both eyes and eyebrows; the mouth region was defined using a rectangle of 208 × 139 pixels. Images subtended a visual angle of 10°. Results of total fixation counts, and absolute dwell times, within these pre-determined ROI are reported. It was assumed that both the total dwell time and fixation count reflects a combination of participants' interest in, and attraction to, the information within the ROI and how relevant they found this information for categorizing the expressions. By measuring both dwell time and fixation count we aimed to see if a relationship of psychopathic traits with eye scan paths for emotional faces can be observed across different eye tracking parameters. No time limits were imposed on participants' responses for each trial.

In order to examine differences in eye tracking parameters recorded using the tower and head mounted eye tracking techniques, we computed mixed model ANCOVAs, including emotion, intensity (mild, moderate, high), sex, and ROI, with the eye tracking technique included as a covariate. Analyses for both fixation counts and total dwell time revealed no significant interactions of the eye tracking technique used. Thus, analyses including psychopathic traits were collapsed across the two techniques. Mixed ANCOVAs, as described above, were used to analyze absolute dwell time, and total fixation counts, focusing on effects that interacted with psychopathic traits. Interactions with psychopathic traits were broken down using both zero-order and partial correlations.

## Results

### Psychopathic traits

Scores on the primary psychopathy scale ranged from 20 to 48 (*M* = 28.9, *SD* = 6.2) out of a possible 64, while secondary psychopathy scores ranged from 14 to 26 (*M* = 20.5, *SD* = 3.2) out of a possible score of 40. These scores are within the range reported by others for non-offending samples, with primary psychopathy scores in earlier studies found to range from 28 to 35, and secondary psychopathy from 20 to 23 (Levenson et al., 1995; Campbell et al., 2009; Gummelt et al., 2012). Scores on the two subscales were significantly positively correlated (*r* = 0.50, *p* = 0.002).

### Accuracy

An ANCOVA on accuracy revealed a significant effect of intensity *F*_(2, 48)_ = 53.72, *p* < 0.001, $\eta_{p}^{2}=0.69$, with expressions at lower intensity recognized with a lower degree of accuracy. Bonferroni adjusted pairwise comparisons revealed that high intensity expressions were recognized with greater accuracy than moderate intensity ones, and moderate intensity expressions were recognized more accurately than mild intensity ones (all *p* < 0.001) (see Table 1). The low accuracy for mild intensity expressions likely reflects the fact that these expressions contain only 10% of the emotionally expressive information, and were therefore predominantly (84.5%) judged to be neutral. There were no other main effects or interactions, nor were there any effects of primary or secondary psychopathic traits (all *p* > 0.05). Using zero-order and partial correlations, we found no relationship of scores on the primary or secondary psychopathy scales with recognition accuracy for negative expressions (angry, disgust, fear, or sad) at moderate or high intensity (all *p* > 0.05).

**Table 1:** **Accuracy (% correct) of emotional expression recognition by expression and intensity, collapsed across sex of model**.

**Intensity**	**% Correct ***M***(***SD***)**					
	**Anger**	**Disgust**	**Fear**	**Happy**	**Sad**	**Surprise**
Low (10%)	1.3 (4.3)	0.3 (1.8)	5.0 (9.0)	0 (0)	9.3 (16.2)	4.0 (7.2)
Moderate (55%)	85.3 (19.1)	78.0 (19.2)	69.0 (27.1)	94.0 (19.6)	75.3 (20.4)	87.0 (19.0)
High (90%)	88.3 (18.6)	88.3 (19.8)	69.3 (25.9)	96.3 (18.3)	85.3 (18.1)	82.7 (20.8)

### Eye tracking

**Figure 2:**
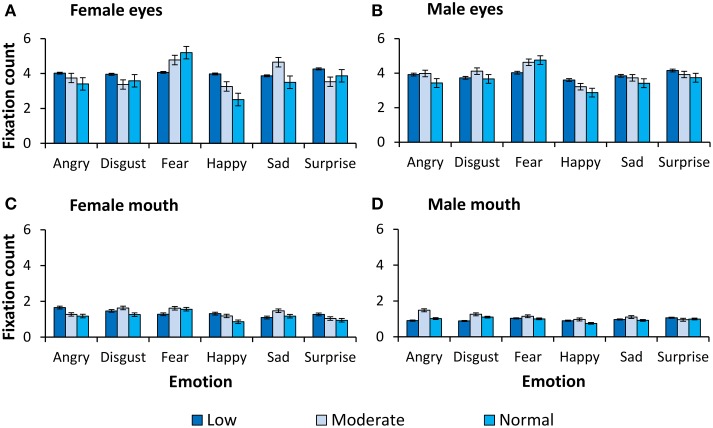
**Number of fixations on the eyes and the mouth**. Fixation counts as a function of expression and intensity for **(A)** female eyes, **(B)** male eyes, **(C)** female mouth, and **(D)** male mouth.

**Figure 3:**
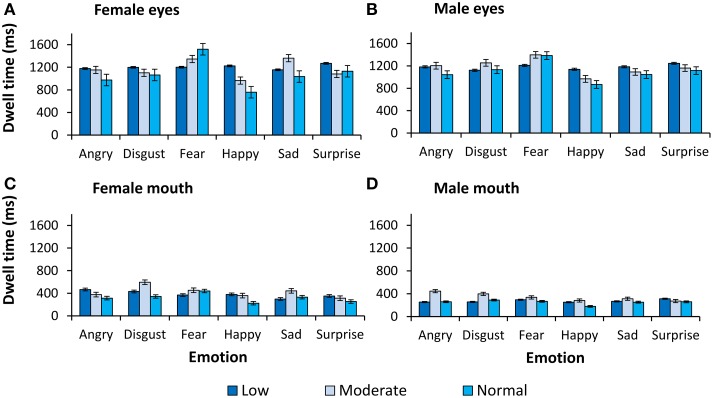
**Dwell time (ms) on the eyes and the mouth**. Dwell time (ms) as a function of expression and intensity for **(A)** female eyes, **(B)** male eyes, **(C)** female mouth, and **(D)** male mouth.

For completeness, all observed main effects and interactions, excluding interactions with primary or secondary psychopathic traits, are reported in Table 2. In Figures 2, 3 we show fixation counts and dwell times, respectively, on the eyes and the mouth as a function of the emotion expressed, and the intensity and sex of the expression.

**Table 2:** **Summary of significant main effects and interactions for analyses of fixation counts and dwell time, excluding interactions with psychopathic traits**.

**Effect**	***F***	**df**	***p***	${\text{η}}_{p}^{2}$
**FIXATION COUNT**				
ROI	6.85	1, 34	0.01	0.17
Emotion × Sex × ROI	3.28	5, 170	0.01	0.09
**DWELL TIME**				
ROI	7.53	1, 34	0.01	0.18
Emotion × ROI	3.27	5, 170	0.01	0.09
Emotion × Sex × ROI	2.28	5, 170	0.05	0.06
Level × Sex × ROI	3.57	2, 68	0.03	0.10

### Effects of psychopathic traits on fixation count

**Figure 4:**
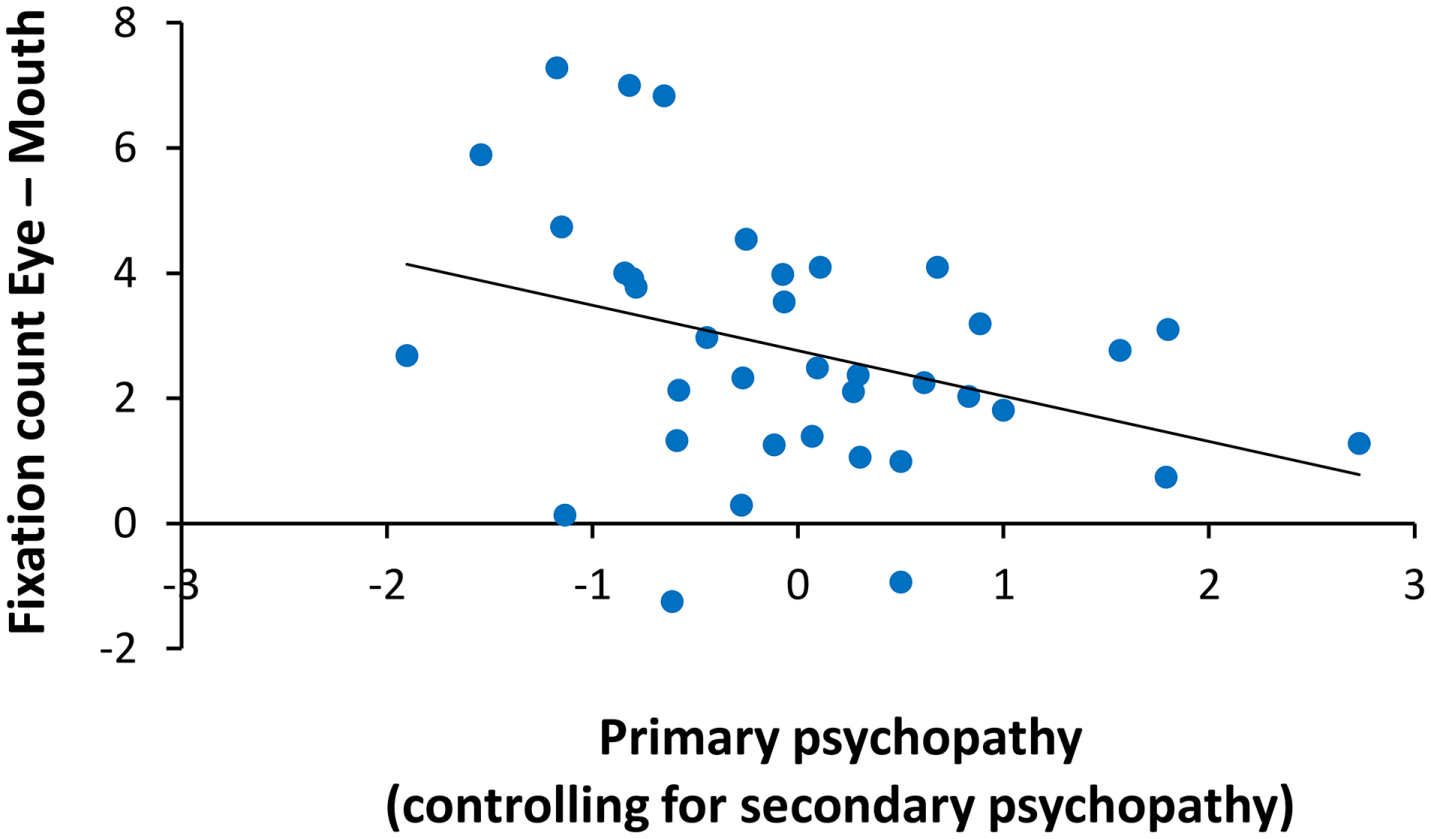
**Relationship of primary psychopathic traits with fixation count on the eyes relative to the mouth**. When controlling for secondary psychopathic traits, primary psychopathic traits were associated with fewer fixations on the eyes relative to the mouth (eyes–mouth) across expressions, intensities, and sex (*r* = −0.36, *p* = 0.03).

An ANCOVA on fixation count with primary and secondary psychopathic traits as covariates revealed a significant interaction of ROI with primary psychopathic traits *F*_(1, 34)_ = 4.99, *p* = 0.03, $\eta_{p}^{2}=0.13$. A partial correlation of primary psychopathic traits with the relative number of fixations on the eyes compared to the mouth (eye–mouth), across all trials and controlling for secondary psychopathic traits, showed a negative relationship (*r* = −0.36, *p* = 0.03). The zero-order correlation of primary psychopathic traits with number of fixations on the eyes relative to the mouth was similarly significant (*r* = −0.37, *p* = 0.03). Here we showed that increasing levels of primary psychopathic traits were associated with fewer fixations on the eyes relative to the mouth (see Figure 4). There were no other significant interactions with primary or secondary psychopathic traits.

### Effects pf psychopathic traits on dwell time

**Figure 5:**
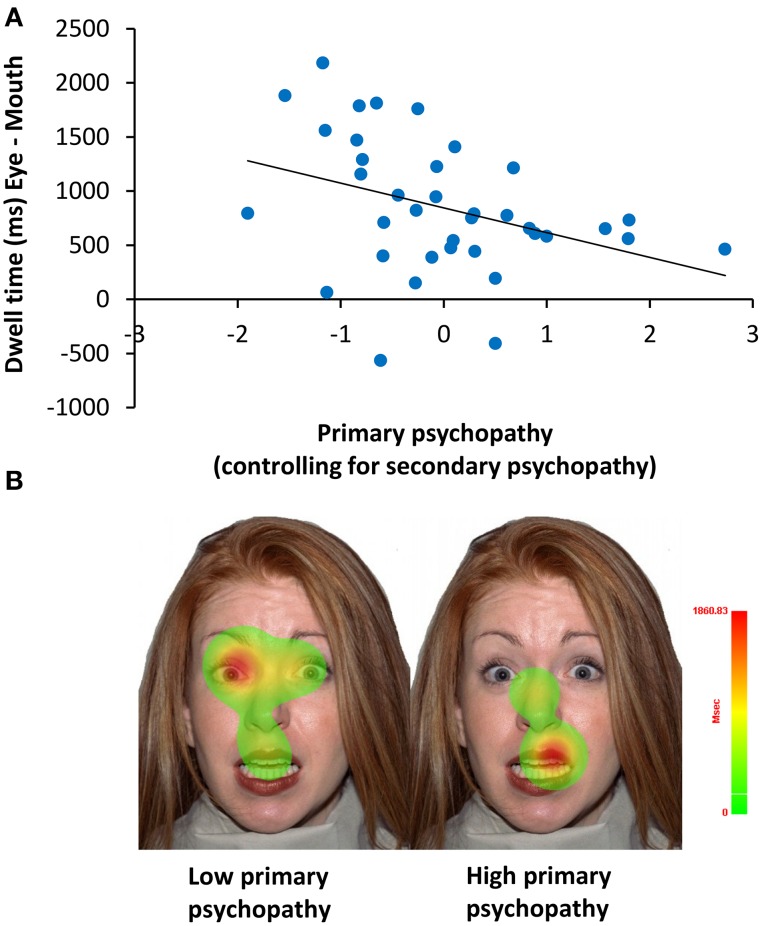
**Relationship of primary psychopathic traits with dwell time on the eyes relative to the mouth. (A)** When controlling for secondary psychopathic traits, primary psychopathic traits were associated with shorter dwell time (ms) on the eyes relative to the mouth (eyes–mouth) across expressions, intensities, and sex (*r* = −0.37, *p* = 0.03). **(B)** Heat maps showing attention to the eyes and the mouth for a single low scoring (Left) and a single high scoring (Right) primary psychopathic traits participant.

A mixed model ANCOVA on dwell time also revealed a significant interaction of ROI with primary psychopathic traits *F*_(1, 34)_ = 5.36, *p* = 0.03, $\eta_{\text{p}}^{2}=0.14$. To better understand this interaction, we computed a partial correlation of primary psychopathic traits, controlling for secondary psychopathic traits, with dwell time on the eyes relative to the mouth (eye–mouth) across all trials. This analysis showed that increasing levels of primary psychopathic traits were associated with reduced dwell time on the eyes compared to the mouth of emotionally expressive faces (*r* = −0.37, *p* = 0.03) (see Figure 5). A similar result was also obtained using a zero-order correlation (*r* = −0.39, *p* = 0.02).

We also revealed significant interactions of intensity and sex with primary psychopathic traits *F*_(2, 68)_ = 3.43, *p* = 0.04, $\eta_{{\text{p}}^{2}}=0.09$, emotion and ROI with primary psychopathic traits *F*_(5, 170)_ = 2.35, *p* = 0.04, $\eta_{{\text{p}}^{2}}=0.07$, and intensity, sex, and ROI with primary psychopathic traits *F*_(2, 68)_ = 3.79, *p* = 0.03, $\eta_{{\text{p}}^{2}}=0.10$. However, there was a significant higher order interaction of emotion, intensity, sex, and ROI with primary psychopathic traits *F*_(10, 340)_ = 2.34, *p* = 0.01, $\eta_{{\text{p}}^{2}}=0.06$. The presence of a higher order interaction suggests that, the relationship of primary psychopathic traits with attention to the eyes relative to the mouth, may be most pronounced for particular emotions, though these effects are dependent upon the intensity and sex of the expression. Thus, when broken down by emotion expressed, we found a significant interaction of intensity, sex, and ROI with primary psychopathic traits for expressions of anger *F*_(2, 68)_ = 10.35, *p* < 0.001, $\eta_{{\text{p}}^{2}}=0.23$, and fear *F*_(2, 68)_ = 3.18, *p* < 0.05, $\eta_{{\text{p}}^{2}}=0.09$. The interactions of intensity, sex, and ROI with primary psychopathic traits for expressions of disgust, happiness, sadness, and surprise were all non-significant. Next, we carried out further analyses to better understand the interactions of intensity, sex, and ROI with primary psychopathic traits for angry and fearful expressions.

For angry expressions broken down by intensity, we observed significant interactions of sex and ROI with primary psychopathic traits for moderate intensity *F*_(1, 34)_ = 10.37, *p* = 0.003, $\eta_{{\text{p}}^{2}}=0.23$, and high intensity expressions *F*_(1, 34)_ = 11.45, *p* = 0.002, $\eta_{{\text{p}}^{2}}=0.25$. When broken down by sex, we observed a significant interaction of ROI with primary psychopathic traits for male expressions at moderate intensity *F*_(1, 34)_ = 5.00, *p* = 0.03, $\eta_{{\text{p}}^{2}}=0.13$, and for female expressions at high intensity *F*_(1, 34)_ = 9.40, *p* = 0.004, $\eta_{{\text{p}}^{2}}=0.22$. A partial correlation of primary psychopathic traits, with dwell time on the eyes compared to the mouth for angry male expressions at moderate intensity, revealed a significant negative correlation (*r* = −0.36, *p* = 0.03). A zero-order correlation was also similarly significant (*r* = −0.39, *p* = 0.02). We also showed a similar result for angry female expressions at high intensity, with primary psychopathic traits inversely correlated with dwell time on the eyes relative to the mouth when controlling for secondary psychopathic traits (*r* = −0.47, *p* = 0.004). Again, the zero-order correlation was also significant (*r* = −0.46, *p* = 0.004). Thus, for angry expressions, we showed that primary psychopathic traits were associated with reduced dwell time on the eyes compared to the mouth for moderate intensity male expressions, and high intensity female expressions.

For fearful faces broken down by intensity, we observed a significant interaction of sex and ROI with primary psychopathic traits for expressions at high intensity only *F*_(1, 34)_ = 6.22, *p* = 0.02, $\eta_{{\text{p}}^{2}}=0.16$. We further broke this interaction down by sex and showed that there was a significant interaction of ROI with primary psychopathic traits for female expressions only *F*_(1, 34)_ = 6.85, *p* = 0.01, $\eta_{{\text{p}}^{2}}=0.17$. A partial correlation controlling for secondary psychopathic traits showed that there was a significant inverse relationship of primary psychopathic traits with dwell time on the eyes relative to the mouth for fearful female expressions at high intensity (*r* = −0.41, *p* = 0.01), while the zero-order correlation was also significant (*r* = 0.45, *p* = 0.01).

Thus, for analyses of dwell time, we showed that across emotion, intensity, and sex, there was a significant inverse relationship of primary psychopathic traits with dwell time on the eyes compared to the mouth. Furthermore, this relationship was particularly pronounced for angry male expressions at moderate intensity, angry female expressions at high intensity, and female fearful expressions at high intensity.

### Association of eye scan paths and accuracy

Finally, we examined the relationships between eye scan paths and accuracy of expression recognition. It was predicted *a-priori* that accuracy for negative emotional expressions (anger, disgust, fear, sadness) would be related to differences in fixation counts and dwell time on the eyes and the mouth. A correlational analysis of fixation counts on the eyes relative to the mouth (eye–mouth) for high intensity fear expressions showed that a greater number of fixations on the eye region, relative to the mouth, was associated with more accurate performance for fearful expressions (*r* = 0.37, *p* = 0.04). Furthermore, we also identified a trend for angry faces (*r* = 0.34, *p* = 0.07), with higher fixation counts for the eyes, relative to the mouth, again found to be positively correlated with accuracy of angry expression recognition at high intensity. We found no significant relationships between dwell time parameters and accuracy of expression recognition at either moderate or high intensity, for anger, disgust, fear, or sad faces (all *p* > 0.09).

## Discussion

The aim of this experiment was to investigate the association of primary and secondary psychopathic traits with attention to the eye region in a sample of non-offending adult males. Also, we aimed to investigate the effects of the emotional content of the face, and the intensity and sex of the expression. We showed that primary psychopathic traits were associated with reduced attention to the eye region relative to the mouth. This finding was observed across male and female expressions of the six basic emotions, at varying levels of intensity. Furthermore, these findings held for both total fixation count and overall dwell time, and were independent of secondary (lifestyle/antisocial) psychopathic traits. These findings are the first to extend the finding of psychopathy related impairments in the directing of attention to the eyes of others emotional expressions to an adult male sample.

Although it was predicted that psychopathic traits would be inversely associated with accuracy for negative emotions, in particular fear, we failed to observe a relationship of accuracy with either primary or secondary psychopathic traits. These findings are in contrast to results showing impaired recognition of emotional faces among adult male psychopaths (Blair et al., 2004), and among children with CU traits (Blair et al., 2001; Dadds et al., 2008). However, these results may reflect the subclinical nature of the current sample. For example, Gordon et al. (2004) also report a failure to identify behavioral differences in emotion recognition between non-offenders scoring high and low on a self-report measure of psychopathy, despite finding a differential neural response in the amygdala and areas of the pre frontal cortex. Thus, although we observed reduced attention to the eyes in relation to psychopathic traits, this reduction may not have been sufficient to adversely affect participants' emotion recognition. Alternatively, these contrasting results may reflect methodological differences between the current and previously published studies. Previous studies with adult male psychopaths have shown faces that morph from neutral to expressive in successive frames (Blair et al., 2004), or have imposed viewing time constraints in developmental samples (Dadds et al., 2008). In contrast, the present study allowed unlimited viewing time of a single emotional expression image per trial.

For analyses of fixation count and dwell time, we showed that there was a significant interaction of primary psychopathic traits with attention to the eyes relative to the mouth. This effect was consistent with our hypotheses and showed that increasing levels of primary psychopathic traits were associated with a reduced number of fixations, and lower levels of overall dwell time, on the eyes relative to the mouth. These findings are consistent with those of Dadds et al. (2008) who found an inverse correlation of CU traits with the length and number of fixations on the eyes of emotionally expressive faces in a developmental sample. Taken together, these findings suggest that psychopathic traits in both child and adolescent samples, and adult non-offender samples, are associated with reduced attention to the eye region of emotional faces.

Analyses of dwell time also showed that primary psychopathic traits were associated with reduced attention to the eyes, compared with the mouth, for angry male expressions at moderate intensity, angry female expressions at high intensity, and female fear expressions at high intensity. Effects of psychopathy for anger and fear expressions suggest that impaired attention to the eyes may be most pronounced where the eyes are particularly diagnostic of the emotional content of the expression. Smith et al. (2005) showed that information from the eyes was used with high optimality, or was used with the greatest efficiency, for the categorization of both angry and fearful expressions. Furthermore, Dadds et al. (2008) found positive correlations of fear accuracy with both the number, and length, of fixations on the eye region. Information from the eye region may therefore be of particular importance for the accurate classification of angry and afraid faces, and a failure to fixate the eye region of these faces may result in particularly impaired performance in judging these emotions. Findings from the present study appear to support this interpretation, with positive correlations observed for anger and fear accuracy with fixation counts on the eyes relative to the mouth for high intensity expressions.

It should be noted that while impairments in directing attention to the eyes have also been observed among children with Autism Spectrum Disorders [ASDs] (Pelphrey et al., 2005; Jones and Klin, 2013), the precise mechanisms underlying these difficulties are likely to differ in psychopathy and autism (Blair, 2008; Gillespie et al., 2014). This postulated difference in mechanisms is supported, for example, by observations that children with ASD, but not those with CU traits, show atypical neural processing associated with theory of mind (O'Nions et al., 2014). However, children with CU traits, but not those with ASD, show difficulties in resonating with other people's emotions (Jones et al., 2010). Blair (2003) argues that psychopaths' lack of empathy may reflect abnormal amygdala function, with amygdala based accounts of the disorder focusing on the role of the amygdala in the ability to recognize and learn from other peoples distress cues. In support of such models, we would note that reduced attention to the eyes, as reported here in relation to primary psychopathic traits, has also been observed among patients with bilateral amygdala dysfunction (Adolphs et al., 2005).

The findings reported here, and the similar findings of Dadds et al. (2006, 2008), may have implications for intervening in psychopathy. For example, a simple instruction to fixate the eye region of emotional faces has been shown to lead to temporary correction of fear recognition impairments among patients with bilateral amygdala dysfunction (Adolphs et al., 2005), and among children with conduct problems and CU traits (Dadds et al., 2006, 2008). Furthermore, improvements in affective empathy and conduct problems have also been observed following expression recognition training in children referred for emotional and behavioral problems (Dadds et al., 2012). However, the long term effects of expression recognition training in adult male psychopaths remain unknown.

Similarly, the mechanisms underlying the initiation of eye movements toward the eye region also remain unclear. In one study it has been shown that the amygdala may be involved in reflexive shifts of attention toward the eyes (Gamer and Büchel, 2009), and it is noted by Vuilleumier (2015) that the amygdala assigns emotional salience to features in the environment, can modulate enhancement of visual perception, and facilitate orienting of attention and eye gaze toward emotionally salient information. Thus, a failure of the amygdala to assign emotional significance to the eye region of emotional faces may contribute to impaired visual attention to the eyes in psychopathy. Alternatively, reduced attention to the eyes may result from more generally impaired orienting mechanisms in relation to CU traits (Dawel et al., 2015). Reduced attention to the eyes may also have implications for neuroimaging studies in samples with psychopathic tendencies. Although hypoactivity of the amygdala in response to emotional faces has been noted in relation to psychopathy, these findings may reflect differences in the attended to regions of the face, with lesser attention to the eyes expected among those scoring more highly for psychopathic tendencies (Dadds et al., 2008).

The current findings may be subject to certain methodological limitations, including the use of self-report measures of psychopathy that may be vulnerable to deception. However, although a deceitful and manipulative interpersonal style is recognized as one of the defining features of psychopathic personality (Lilienfeld and Fowler, 2006), results from a recent meta-analysis suggest that self-report measures nonetheless represent a reliable means of assessing psychopathic traits (Ray et al., 2013). Also, a low prevalence of psychopathy has been reported in the general household population of Great Britain (Coid et al., 2009), and it is likely that participants in the current study showed only mild levels of psychopathic features. Psychopathic trait levels for the current sample were in the normative range of values reported by others for the primary and secondary subscales of the LSRP (Levenson et al., 1995; Campbell et al., 2009; Gummelt et al., 2012). However, given the low levels of psychopathy in the general population, the finding of reduced attention to the eyes in relation to psychopathy should also be replicated in samples of offending participants, or participants with clinical levels of psychopathy.

The findings reported here are also unrevealing about the relation of psychopathy with attention to the eyes in female participants, or across different ethnic groups. Psychopathic traits are thought to manifest differently in male and female participants (Sprague et al., 2012), and the extent to which these variations affect eye scan paths for emotional faces remains unknown. Also, although the factor structure of psychopathy has been shown to be invariant across ethnicity in both clinical (Cooke et al., 2001), and community samples (Neumann and Hare, 2008), non-white participants in the general household population of Great Britain show higher scores across different psychopathic traits compared to white participants (Coid et al., 2009). Thus, our results from a majority Caucasian, adult male sample do not shed light on potential differences in the association of psychopathy and attention to the eyes across different ethnic groups. Finally, although participants were tested using either a head mounted or a towered eye tracking system, we found no effect of the eye tracking technique used on effects for either fixation count or dwell time.

## Conclusion

The findings reported here support the hypothesis that psychopathic traits are associated with reduced attention to the eye region, and confirm the results from developmental samples in a non-offending sample of adult males. The finding of reduced attention to the eyes was observed for both the total number of fixations, and overall dwell time, and was independent of secondary (lifestyle/antisocial) psychopathic traits. Although participants in this study did not show a psychopathy related impairment in emotion recognition, similar impairments in forensic and clinical samples may be explained by reduced attention to the emotionally significant aspects of the face. These hypotheses should be tested in appropriate forensic/clinical adult samples. The results of the current study also appear to be consistent with amygdala based accounts of psychopathy, with similarities in the findings reported here and those reported among neuropsychological patients with amygdala dysfunction. However, the relationship of amygdala function with reduced attention to the eyes remains somewhat unclear. Further, these findings raise important issues regarding the interpretation of findings that psychopaths show reduced amygdala response while viewing facial emotional expressions. As noted by Dadds et al. (2008), these findings may reflect the tendency for high psychopathic traits participants to focus attention on different aspects of the face, most notably the eye region. This issue should be resolved in future research investigating the neural correlates of emotional face processing in psychopathy.

### Conflict of interest statement

The authors declare that the research was conducted in the absence of any commercial or financial relationships that could be construed as a potential conflict of interest.
